# Concurrent Chemoradiotherapy Increases the Levels of Soluble Immune Checkpoint Proteins in Patients with Locally Advanced Cervical Cancer

**DOI:** 10.1155/2022/9621466

**Published:** 2022-04-04

**Authors:** Chao Liu, Xiaohui Li, Aijie Li, Wenxue Zou, Rui Huang, Xiaoyu Hu, Jinming Yu, Xiaoling Zhang, Jinbo Yue

**Affiliations:** ^1^Cheeloo College of Medicine, Shandong University, Jinan 250012, China; ^2^Department of Radiation Oncology, Shandong Cancer Hospital and Institute, Shandong First Medical University and Shandong Academy of Medical Sciences, Jinan 250117, China; ^3^Department of Gynecologic Oncology, Shandong Cancer Hospital and Institute, Shandong First Medical University and Shandong Academy of Medical Sciences, Jinan 250117, China

## Abstract

**Purpose:**

Concurrent chemoradiotherapy (CCRT) has been widely applied to locally advanced cervical cancer (LACC) patients, inducing the massive release of antigen and systematic immunomodulatory effects. However, its effect on the soluble immune checkpoint proteins (sICPs) remains unclear, which might play a key role in the immune response. Therefore, the current study explored changes in the levels of 16 sICPs in LACC patients during CCRT.

**Methods:**

We prospectively enrolled fifty-one LACC patients treated with CCRT and collected patients' blood before, during and after CCRT. The levels of 16 sICPs were measured using the Luminex platform, and the changes were measured using Friedman test with Bonferroni's posttest. One month after CCRT, the tumor response was evaluated according to the RECIST 1.1 guidelines.

**Results:**

The levels of soluble T-cell immunoglobulin and mucin-domain containing-3 (sTIM-3) significantly increased during CCRT (*P* = 0.041), while those of the soluble B and T lymphocyte attenuator (sBTLA), sCD40, soluble glucocorticoid-induced tumor necrosis factor receptor ligand (sGITRL), sCD80, sCD86, sPD-1, sPD-L1, sCTLA-4, and soluble inducible T-cell costimulator (sICOS) significantly increased after CCRT (all *P* < 0.05). Other sICPs showed no significant changes throughout the CCRT (all *P* > 0.05). 41 (80%), 8 (16%), and 2 (4%) patients showed complete response (CR), partial response (PR), and stable disease (SD) after CCRT, respectively. Interestingly, the level of soluble lymphocyte-activation gene 3 (sLAG-3) was significantly higher among the PR/SD patients as compared to the CR after CCRT (*P* = 0.009).

**Conclusions:**

This study revealed that CCRT might elevate the serum levels of sTIM-3, sBTLA, sCD40, sGITRL, sCD80, sCD86, sPD-1, sPD-L1, sCTLA-4, and sICOS in the patients with LACC. The sLAG-3 level was higher in the patients with poor response to CCRT. These findings revealed the dynamic changes in the sICPs levels during CCRT, which might be helpful in designing optimal treatment strategies for LACC patients.

## 1. Introduction

Concurrent chemoradiotherapy (CCRT) is currently a standard therapy for locally advanced cervical cancer (LACC) as per the recommendations of the National Comprehensive Cancer Network [1]. The recent 5-year survival rate of the LACC patients treated with CCRT is approximately 71%, while 23% of the patients still experience local or metastatic relapses after CCRT [2]. Therefore, the extensive effects of CCRT on LACC patients have been attaching more attention to achieve better survival.

In recent years, an increasing number of studies have supported the notion that radiation not only directly kills tumor cells by ionizing radiation but also triggers a local and systemic immune response by releasing the tumor antigen [3–6]. Previous studies have advanced the understanding of how CCRT regulates the activation of systemic immune by analyzing the immune checkpoints of tumor biopsy and T lymphocyte populations in peripheral blood mononuclear cells (PBMCs). CCRT can modify the tumor immune microenvironment by reducing the PD-1/PD-L1 expression and upregulating the CD28 costimulation signal [7, 8]. Besides, Li et al. found the increasing number of CD4^+^ and CD8^+^ T cells and the decreasing number of inhibitory regulatory T cells in PBMCs after CCRT [8]. However, to the best of our knowledge, the serum levels of immune checkpoints during CCRT have not been studied among the LACC patients yet. The soluble immune checkpoint proteins (sICPs) are generated from the mRNA expression or cleavage of membrane-bound proteins and play a key role in the immune regulation and escape [9–12]. Therefore, there is an urgent need to thoroughly explore the changes in the sICPs levels during the whole CCRT.

In the present study, the serum levels of 16 sICPs were measured among the paired plasma samples of 51 LACC patients before, during, and after CCRT using the Luminex platform. The results showed alterations in the patterns of sICPs throughout CCRT and also identified key sICP involved in the pathological response. These results might provide novel insights into the changes in the immune microenvironment throughout CCRT and more evidence for a novel therapeutic target for the LACC patients.

## 2. Methods

### 2.1. Study Design and Patients

This study was approved by the Ethical Committee of Shandong Cancer Hospital and Institute. All patients presented written informed consents before enrolment. The participants were diagnosed with cervical cancer at the Federation International of Gynecology and Obstetrics (FIGO) stage IB2 to IVA using biopsy in Shandong Cancer Hospital and Institute. One month after CCRT, the tumor response was assessed as per RECIST 1.1 guidelines.

The inclusion criteria for the recruitment of patients were pathological diagnosis, no previous history of antitumor therapy or immunodeficiency disease, ≥18 years of age, and performance status ≤1 (Eastern Cooperative Oncology Group score). A total of 56 patients were enrolled from July 2017 to May 2019; among which, five patients were excluded based on the inclusion criteria. The remaining 51 patients received the designed CCRT.

### 2.2. Treatment Methods

Radiotherapy (RT) included pelvic external beam radiation therapy of 45-50.4 Gy in 25-28 fractions and brachytherapy of 30-40 Gy in 6-8 fractions. Concurrent chemotherapy of paclitaxel (175 mg/m^2^) followed with cisplatin (30-40 mg/m^2^) or carboplatin (dosed to an area under the curve of 5 to 6) was administered every 3 weeks for up to two cycles of RT.

### 2.3. Clinical Data

The baseline characteristics of patients included age, sex, smoking history, tumor size, squamous cell carcinoma (SCC) antigen, human papillomavirus (HPV) infection status, histological types, primary tumor (T) stage, node (N) stage, and FIGO stage.

### 2.4. Sample Collection and Measurement of sICPs

The patients' blood samples were collected before, during (3-4 weeks after the initiation of RT), and after CCRT (approximately 1 week after the completion of RT). After centrifuging for 10 min at 2000 rpm, plasma was obtained from the blood samples, stored in a refrigerator at −80°C, and thawed to room temperature (20-25°C) before the measurement of sICPs. The plasma levels of the 16 sICPs were measured using the MILLIPLEX® Human Immuno-Oncology Checkpoint Protein Panel1 (Cat. # HCKPMAG-11 K; Millipore) following the manufacturer's instructions. Then, the assays were read using the Luminex platform to determine the final levels of the samples.

### 2.5. Statistics Analyses

Differences in the levels of sICPs among the 3 groups were analyzed using the Friedman test with Bonferroni's posttest. Differences in the levels of sICPs between the CR and PR/SD groups were analyzed using the Kolmogorov-Smirnov test. All the data are presented as mean ± SEM.

## 3. Results

### 3.1. Patients' Characteristics

The baseline characteristics of the LACC patients are listed in Table 1. A total of 51 patients (median age, 52 years; range, 30-77 years) were included in this study. All the patients (100%) had SCC, which was confirmed by pathology. Based on the FIGO stage, 16 (31%) patients were at stage IB-IIB, while 35 (69%) patients were at stage IIIB-IVA. The lymph node metastasis was positive in 34 patients (59%). The tumor median volume was 5.1 cm, ranging from 2.4 to 7.6 cm. Among the 51 patients, 49 (97%) patients had HPV infections. One month after CCRT, 41 patients (80%) showed complete response (CR), 8 patients (16%) showed partial response (PR), and 2 patients (4%) showed stable disease (SD).

### 3.2. Changes in the sICPs Levels during CCRT

**Figure 1:**
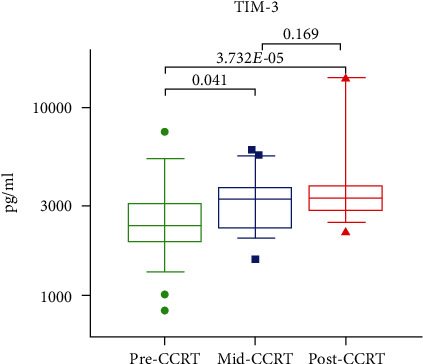
Significant changes in the sTIM-3 level during CCRT. The sTIM-3 levels were compared among pre-CCRT (green), mid-CCRT (blue), and post-CCRT (red), showing significant changes during CCRT. The *P* values were calculated using the Friedman test with Bonferroni's post-test.

First, the changes in the levels of sICPs during CCRT were investigated by analyzing the sICPs levels in the LACC patients before and during CCRT. Ultimately, a significant increase in the median serum levels of T-cell immunoglobulin and mucin-domain containing-3 (sTIM-3) (*P* = 0.041) was observed as compared to the baseline. However, no significant change was observed in the levels of other sICPs (Figure 1).

### 3.3. Changes in the sICPs Levels after CCRT

**Figure 2:**
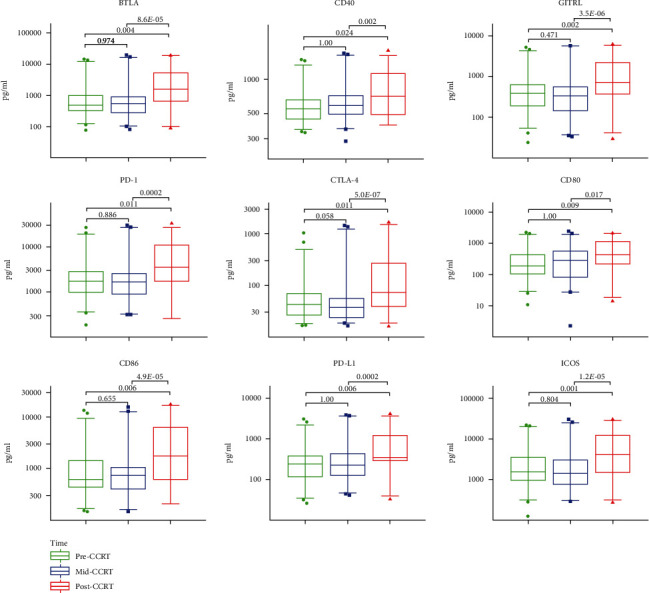
Significant changes in the sICPs after CCRT. The serum levels of BTLA, CD40, GITRL, PD-1, CTLA-4, CD80, CD86, PD-L1, and ICOS were compared among pre-CCRT (green), mid-CCRT (blue), and post-CCRT (red), showing significant changes after CCRT. *P* values were calculated using the Friedman test with Bonferroni's post-test.

Next, the changes in the sICPs levels after CCRT were investigated by analyzing the sICPs levels in the LACC patients during and after CCRT. Eventually, a significant increase in the median levels of soluble B and T lymphocyte attenuator (sBTLA), sCD40, soluble glucocorticoid-induced tumor necrosis factor receptor ligand (sGITRL), sCD80, sCD86, sPD-1, sPD-L1, sCTLA-4, and soluble inducible T-cell costimulator (sICOS) was observed after CCRT. Their levels were significantly higher than their respective levels before and during CCRT (all *P* < 0.05) (Figure 2).

**Figure 3:**
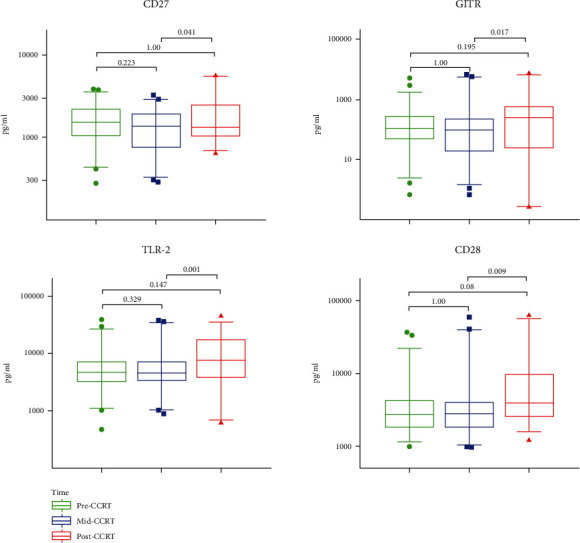
Slight changes in the levels of sICPs after CCRT. The serum levels of CD27, GITR, TLR-2, and CD28 were compared among pre-CCRT (green), mid-CCRT (blue), and post-CCRT (red), showing slight changes after CCRT. No significant difference was found in the levels between pre-CCRT and post-CCRT. The *P* values were calculated using the Friedman test with Bonferroni's post-test.

**Figure 4:**
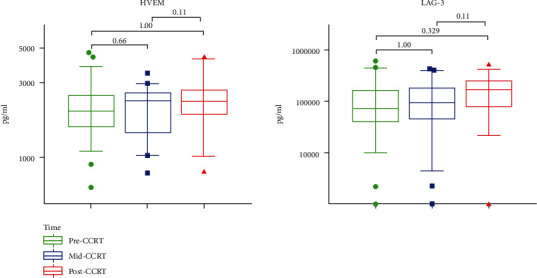
No changes in the levels of sICPs during the CCRT. The serum levels of HVEM and LAG-3 were compared among pre-CCRT (green), mid-CCRT (blue), and post-CCRT (red). *P* values were calculated using the Friedman test with Bonferroni's post-test.

Besides, the median levels of sGITR, toll-like receptor (sTLR-2), and sCD28 slightly increased after CCRT, which were significantly higher than their levels during CCRT (*P* = 0.017, 0.001, and 0.009, respectively), but had no difference as compared to the baseline. Additionally, CD27 was the only factor, which showed a significant decrease after CCRT (*P* = 0.041), but had no difference as compared to its level before CCRT (Figure 3). Moreover, the median levels of soluble herpesvirus entry mediator (sHVEM) and lymphocyte-activation gene-3 (sLAG-3) showed no differences throughout CCRT (Figure 4).

### 3.4. Differences in the sICPs Levels between the CR and PR/SD Groups

**Figure 5:**
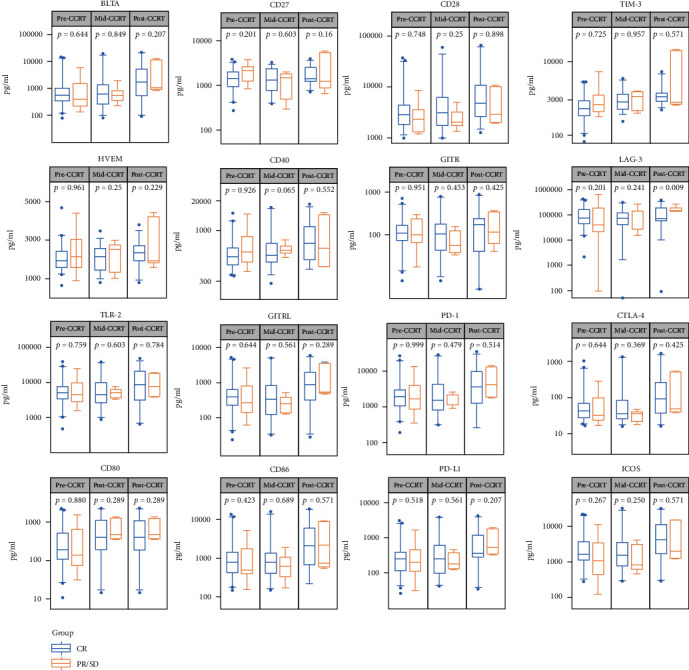
Differences in the sICPs between the CR and PR/SD groups patients. The level of sICPs was compared between the CR and PR/SD group patients before, during, and after CCRT. The *P* values were calculated using the Kolmogorov-Smirnov test.

In order to explore the clinical significance of sICPs, the patients were divided into two groups, including CR and PR/SD groups. The sICP levels were compared between the two groups before, during, and after CCRT. Interestingly, the median level of sLAG-3 in the PR/SD group patients was much higher as compared to that of the CR group patients after CCRT (*P* = 0.009, nearly twice that of the CR group). Moreover, no significant differences were found in the levels of other sICPs (Figure 5).

## 4. Discussion

In this study, the levels of 16 sICPs were measured in the LACC patients before, during, and after CCRT. The results showed that the plasma level of TIM-3 increased significantly during CCRT, while those of sBTLA, sCD40, sGITRL, sPD-1, sCTLA-4, sCD80, sCD86, sPD-L1, and sICOS increased significantly after CCRT. In addition, a significant difference was also observed in the levels of sLAG-3 between the different pathological response groups of patients. Taken together, the present study revealed different patterns of sICPs levels throughout CCRT, providing evidence for the novel therapeutic targets of LACC treatment.

The ICPs, including stimulatory and inhibitory ICPs, can regulate antigen recognition and T-cell activation/proliferation in the immune response [13]. As previously reported, in the cancer-immunity cycle, CD28: (CD80, CD86), CD40, CD27, HVEM, GITR: GITRL, ICOS, and TLR-2 are stimulatory factors, while TIM-3, PD-L1: PD-1, PD-L1: (CD80, CD86), CTLA-4: (CD80, CD86), BTLA, and LAG-3 are inhibitory factors [13–15]. However, the soluble form of these ICPs might not necessarily have the same positive/negative immune effects as that of the membrane proteins; at present, their function is not fully understood.

In the current study, the levels of sCD28, sCTLA-4, sCD80, sCD86, and sPD-L1 showed a significant increase after CCRT. As mentioned above, if combined with CD28, CD80, and CD86 can provide stimulatory signals, which are required for the T-cell activation and survival, while their interaction with CTLA-4 and PD-L1 might negatively regulate the T-cell response. Some studies have investigated the role of sICPs in cancer-immunity cycle. Kakoulidou et al. [16] reported that recombinant sCD80 could stimulate cytokine production and inhibit T-cell activation and proliferation, thereby suppressing the immune response. They hypothesized that the competitive binding of sCD80 to CTLA-4 might explain the inhibitory response. Besides, a study suggested that the soluble form of CD80 might prevent the apoptotic death of PD-1^+^ activated T cells by neutralizing the PD-L1 or binding to CD28, which might be even more effective than the PD-1 or PD-L1 antibodies [17]. According to the current study results, the increase in the median levels of sCD28, sCTLA-4, sCD80, sCD86, and sPD-L1 might indicate the dual effects of CCRT, increasing both the stimulators and inhibitors.

By interacting with PD-L1, PD-1 can inhibit T cell activation and antitumor immunity in various cancers [18, 19]. It was reported that the coculturing of dendritic cells (DCs) and T cells with sPD-1 could inhibit T cell proliferation, IL-2 production, and DC maturation. They speculated that the binding of sPD-1 and PD-L1 on the DCs might be responsible for the reverse signaling [20]. Moreover, sPD-L1 could exert an inhibitory effect by interacting with membrane-bound PD-1 and other cell surface receptors throughout the body via the blood and lymphatic circulation [21]. In the present study, a persistent increase in the levels of sPD-L1 and sPD-1 after CCRT might reflect the immunomodulatory effect associated with the CD8 + T cell induced by CCRT, which was in line with a previous study [8], revealing that the T cell immunity was markedly suppressed throughout CCRT.

As a member of the TIM family, TIM-3 is a negative costimulatory molecule, which promotes T cell exhaustion in various types of cancer [22]. A study suggested that the competitive binding of sTIM-3 and galectin-9 prevents the inhibitory immune response mediated by TIM-3/galectin-9 [23]. In the present study, an increase in the sTIM-3 during CCRT might indicate the recovery of immune exhaustion.

The prognostic potential of sICPs has been assessed in several studies previously. A study reported that sLAG-3 could induce resistance to Fas-induced and drug-induced apoptosis in the MHC-II-positive melanoma cells. By interacting with MHC-II, sLAG-3 could activate MAPK/Erk and PI3K/Akt pathways, which play a key role in the growth and progression of melanoma [24]. In addition to melanoma, MHC-II is also widely expressed on the surface of HPV-positive cervical cancer cells [25–27]. Interestingly, in this study, sLAG-3 showed a critical role in predicting the pathological response. The median level of sLAG-3 in the PR/SD group patients was nearly twice that of the CR patients after the completion of CCRT, which might be due to the interaction of sLAG-3 and MHC-II in cervical tumor cells.

There are some limitations to this study. First, owing to the limited sample size, some results did not reach statistical significance. Second, the follow-up data of these patients have not been acquired. Furthermore, due to the lack of matching tissue samples, the correlations of these ICPs between tumor tissue and blood could not be identified. Moreover, the mechanism of how these sICPs modulate the tumor immune microenvironment during CCRT remains unclear. Further studies are needed to clarify the specific mechanism.

## 5. Conclusions

In conclusion, this study revealed the dynamic changes in the sICPs levels in LACC patients during CCRT. These results provided new insights into the effects of CCRT on systematic immunity, which might help future studies to develop novel therapeutic approaches and effective combination therapy.

## Figures and Tables

**Table 1:** Baseline characteristics of 51 LACC patients.

Characteristics	*N*	%
Histology (SCC^※^)	51	100
Age (median)	52 (30-77)	/
FIGO stage		
IB-IIB	16	31
IIIB-IVA	35	69
Tumor size (cm)	5.1 (2.4-7.6)	/
Nodal status		
Positive	34	59
Negative	24	41
Smoking history		
Yes	2	4
No	56	96
HPV		
16/18	38	75
Other types	11	22
Negative	2	4
SCC (median, ng/ml)	7.2	/

Data are shown as median (interquartile range) or number (%). Abbreviations: FIGO: Federation International of Gynecology and Obstetrics; HPV: human papillomavirus; SCC^※^: squamous cell carcinoma; SCC: squamous cell carcinoma antigen.

## Data Availability

All data generated or analyzed in this study are included in the published article.

## References

[1] Rose P. G., Bundy B. N., Watkins E. B. (1999). Concurrent cisplatin-based radiotherapy and chemotherapy for locally advanced cervical cancer. *The New England Journal of Medicine*.

[2] Schernberg A., Bockel S., Annede P. (2018). Tumor shrinkage during chemoradiation in locally advanced cervical cancer patients: prognostic significance, and impact for image-guided adaptive brachytherapy. *International Journal of Radiation Oncology • Biology • Physics*.

[3] Herrera F. G., Bourhis J., Coukos G. (2017). Radiotherapy combination opportunities leveraging immunity for the next oncology practice. *CA: a Cancer Journal for Clinicians*.

[4] Chen J., Chen C., Zhan Y. (2021). Heterogeneity of IFN-mediated responses and tumor immunogenicity in patients with cervical cancer receiving concurrent chemoradiotherapy. *Clinical Cancer Research*.

[5] Brix N., Tiefenthaller A., Anders H., Belka C., Lauber K. (2017). Abscopal, immunological effects of radiotherapy: narrowing the gap between clinical and preclinical experiences. *Immunological Reviews*.

[6] Bernstein M. B., Krishnan S., Hodge J. W., Chang J. Y. (2016). Immunotherapy and stereotactic ablative radiotherapy (ISABR): a curative approach?. *Nature Reviews. Clinical Oncology*.

[7] Park S., Joung J. G., Min Y. W. (2019). Paired whole exome and transcriptome analyses for the Immunogenomic changes during concurrent chemoradiotherapy in esophageal squamous cell carcinoma. *Journal for Immunotherapy of Cancer*.

[8] Li R., Liu Y., Yin R. (2021). The dynamic alternation of local and systemic tumor immune microenvironment during concurrent chemoradiotherapy of cervical cancer: a prospective clinical trial. *International Journal of Radiation Oncology • Biology • Physics*.

[9] Li N., Jilisihan B., Wang W., Tang Y., Keyoumu S. (2018). Soluble LAG3 acts as a potential prognostic marker of gastric cancer and its positive correlation with CD8+T cell frequency and secretion of IL-12 and INF-*γ* in peripheral blood. *Cancer Biomarkers*.

[10] Odagiri N., Hai H., Thuy L. T. T. (2020). Early change in the plasma levels of circulating soluble immune checkpoint proteins in patients with unresectable hepatocellular carcinoma treated by lenvatinib or transcatheter arterial chemoembolization. *Cancers (Basel)*.

[11] Machiraju D., Wiecken M., Lang N. (2021). Soluble immune checkpoints and T-cell subsets in blood as biomarkers for resistance to immunotherapy in melanoma patients. *Oncoimmunology*.

[12] Gu D., Ao X., Yang Y., Chen Z., Xu X. (2018). Soluble immune checkpoints in cancer: production, function and biological significance. *Journal for Immunotherapy of Cancer*.

[13] Chen D. S., Mellman I. (2013). Oncology meets immunology: the cancer-immunity cycle. *Immunity*.

[14] He X., Xu C. (2020). Immune checkpoint signaling and cancer immunotherapy. *Cell Research*.

[15] Lim S., Phillips J. B., Madeira da Silva L. (2017). Interplay between immune checkpoint proteins and cellular metabolism. *Cancer Research*.

[16] Kakoulidou M., Giscombe R., Zhao X., Lefvert A. K., Wang X. (2007). Human soluble CD80 is generated by alternative splicing, and recombinant soluble CD80 binds to CD28 and CD152 influencing T-cell activation. *Scandinavian Journal of Immunology*.

[17] Haile S. T., Horn L. A., Ostrand-Rosenberg S. (2014). A soluble form of CD80 enhances antitumor immunity by neutralizing programmed death ligand-1 and simultaneously providing costimulation. *Cancer Immunology Research*.

[18] Sun C., Mezzadra R., Schumacher T. N. (2018). Regulation and function of the PD-L1 checkpoint. *Immunity*.

[19] Butte M. J., Keir M. E., Phamduy T. B., Sharpe A. H., Freeman G. J. (2007). Programmed death-1 ligand 1 interacts specifically with the B7-1 costimulatory molecule to inhibit T cell responses. *Immunity*.

[20] Kuipers H., Muskens F., Willart M. (2006). Contribution of the PD-1 ligands/PD-1 signaling pathway to dendritic cell-mediated CD4+ T cell activation. *European Journal of Immunology*.

[21] Li Y. (2016). Role of soluble programmed death-1 (sPD-1) and sPD-ligand 1 in patients with cystic echinococcosis. *Experimental and Therapeutic Medicine*.

[22] He Y., Cao J., Zhao C., Li X., Zhou C., Hirsch F. (2018). TIM-3, a promising target for cancer immunotherapy. *OncoTargets and therapy*.

[23] Muthukumarana P. A. D. S., Zheng X. X., Rosengard B. R., Strom T. B., Metcalfe S. M. (2008). In primed allo-tolerance, TIM-3-Ig rapidly suppresses TGFbeta, but has no immediate effect on Foxp3. *Transplant international: official journal of the European Society for Organ Transplantation*.

[24] Hemon P., Jean-Louis F., Ramgolam K. (2011). MHC class II engagement by its ligand LAG-3 (CD223) contributes to melanoma resistance to apoptosis. *Journal of immunology (Baltimore, Md. : 1950)*.

[25] Glew S. S., Duggan-Keen M., Cabrera T., Stern P. L. (1992). HLA class II antigen expression in human papillomavirus-associated cervical cancer. *Cancer Research*.

[26] Glew S. S., Connor M. E., Snijders P. J. F. (1993). HLA expression in pre-invasive cervical neoplasia in relation to human papilloma virus infection. *European journal of cancer (Oxford, England: 1990)*.

[27] Zehbe I., Höhn H., Pilch H., Neukirch C., Freitag K., Maeurer M. J. (2005). Differential MHC class II component expression in HPV-positive cervical cancer cells: implication for immune surveillance. *International Journal of Cancer*.

